# Antityrosinase, Antioxidant, and Cytotoxic Activities of Phytochemical Constituents from *Manilkara zapota* L. Bark

**DOI:** 10.3390/molecules24152798

**Published:** 2019-07-31

**Authors:** Sutthiduean Chunhakant, Chanya Chaicharoenpong

**Affiliations:** 1 Program in Biotechnology, Faculty of Science, Chulalongkorn University, Bangkok 10330, Thailand; 2 Institute of Biotechnology and Genetic Engineering, Chulalongkorn University, Bangkok 10330, Thailand; 3 Molecular Crop Research Unit, Faculty of Science, Chulalongkorn University, Bangkok 10330, Thailand

**Keywords:** *Manilkara zapota*, *Sapotaceae*, tyrosinase inhibitor, antioxidant, cytotoxicity

## Abstract

Hyperpigmentation is considered by many to be a beauty problem and is responsible for photoaging. To treat this skin condition, medicinal cosmetics containing tyrosinase inhibitors are used, resulting in skin whitening. In this study, taraxerol methyl ether (**1**), spinasterol (**2**), 6-hydroxyflavanone (**3**), (+)-dihydrokaempferol (**4**), 3,4-dihydroxybenzoic acid (**5**), taraxerol (**6**), taraxerone (**7**), and lupeol acetate (**8**) were isolated from *Manilkara zapota* bark. Their chemical structures were elucidated by analysis of their nuclear magnetic resonance (NMR) spectroscopy and mass spectrometry (MS) data, and by comparing them with data found in the literature. The in vitro antityrosinase, antioxidant, and cytotoxic activities of the isolated compounds (**1**–**8**) were evaluated. (+)-Dihydrokaempferol (**4**) exhibited higher monophenolase inhibitory activity than both kojic acid and α-arbutin. However, it showed diphenolase inhibitory activity similar to kojic acid. (+)-Dihydrokaempferol (**4**) was a competitive inhibitor of both monophenolase and diphenolase activities. It exhibited the strongest 2,2-diphenyl-1-picrylhydrazyl (DPPH), 2,2′-azino-bis(3-ethylbenzothiazoline-6-sulfonic acid (ABTS), and ferric reducing antioxidant power (FRAP) activities of the isolated compounds. Furthermore, (+)-dihydrokaempferol (**4**) also demonstrated potent cytotoxicity in breast carcinoma cell line (BT474), lung bronchus carcinoma cell line (Chago-K1), liver carcinoma cell line (HepG2), gastric carcinoma cell line (KATO-III), and colon carcinoma cell line (SW620). These results suggest that *M. zapota* bark might be a good potential source of antioxidants and tyrosinase inhibitors for applications in cosmeceutical products.

## 1. Introduction

It is well known that free radicals constitute a major risk factor for many diseases, such as cancer, hypertension, asthma, diabetes, and Alzheimer’s disease, as well as aging [1,2]. Antioxidant activity is the most important property of phytochemicals that prevent cellular molecules from oxidative stress. Secondary metabolites of plants are natural antioxidants [3,4]. Antioxidants such as phenolic compounds, flavonoids, and polyphenols trap free radicals and inhibit oxidative stress mechanisms. The oxidation of free radicals can cause the occurrence of melanoma and cancer [5,6]. In the skin, ultraviolet radiation induces the generation of reactive oxygen species (ROS). The ROS mechanism accumulates skin pigmentation on melanocytes. Then, ROS accelerate epidermal phenylalanine hydroxylase (PAH; EC 1.14.16.1). PAH is the rate-limiting enzyme for the production of l-tyrosine. l-Tyrosine is the initial substrate of tyrosinase [7]. Tyrosinase or polyphenol oxidase (EC 1.14.18.1, PPO) is a copper-containing enzyme in melanin biosynthesis [8]. Tyrosinase catalyzes the hydroxylation of l-tyrosine to l-3,4-dihydroxyphenylalanine (l-DOPA) by monophenolase action and the oxidation of l-DOPA to dihydroxyphenylalanine quinone (DOPAquinone) by diphenolase action [9]. The overproduction of pigmentation results in serious aesthetic problems, such as melasma, blemishes, age spots, and freckles [10]. Many skin-whitening agents prevent skin hyperpigmentation through the inhibition of tyrosinase activity. Therefore, tyrosinase inhibitors have been used in skin-whitening products to prevent pigmentation disorders. Kojic acid and α-arbutin are commercial tyrosinase inhibitors that have been used for the treatment of hyperpigmentation [11]. However, they show poor efficacy in vivo, low formulation stability, poor skin penetration, and high toxicity in cells [12]. Thus, the investigation of less toxic and more effective tyrosinase inhibitors is needed. Moreover, ROS may be involved in apoptosis, carcinogenesis, and cell proliferation. ROS induce genetic mutations and damage structural components in cell cycle-related genes [13,14]. Cancer is the second leading cause of death worldwide. The high mortality rate may be an indication of the limited efficiency of cancer therapies such as radiation, chemotherapy, bone marrow transplantation, and surgery [15]. Furthermore, synthetic drugs have been tested for cancer treatment, but their toxicity destroys both tumor cells and normal cells indiscriminately. In addition, the severity of the side effects depends on the therapeutic dose [16,17].

Nevertheless, anticancer properties of medicinal plants have been reported [18,19]. Indeed, anticancer agents currently in clinical use, such as vincristine, vinblastine, paclitaxel, etoposide, and so on, have been isolated from such medicinal plants [20].

*Manilkara zapota* L. (Sapodilla plum) is an evergreen tree with milky juice from the *Sapotaceae* family. Its ripe fruits are edible, possessing a sweet taste [21,22]. *M. zapota* has been reported to exhibit anti-inflammatory, antipyretic, antitumor, antioxidant, antimicrobial, antidiabetic, antilipidemic, anti-aging, and acaricidal activities [23,24,25,26,27,28,29,30,31,32]. Quercitrin and gallic acid have been isolated from *M. zapota* fruits and display antioxidant activity [23]. Myricetin-3-*O*-α-L-rhamnoside has also been isolated from *M. zapota* leaves and exhibits weak antityrosinase activity [33].

In the present study, the tyrosinase inhibitory activity of *M. zapota* bark compounds isolated via a bioactivity-guided process was investigated. The chemical structures of the isolated compounds were identified. The tyrosinase kinetic investigation of the isolated compounds was conducted using Lineweaver–Burk plots. Furthermore, the isolated compounds were evaluated on in vitro antioxidant activity and cytotoxicity against five cancer cell lines—breast carcinoma cell line (BT474), lung bronchus carcinoma cell line (Chago-K1), liver carcinoma cell line (HepG2), gastric carcinoma cell line (KATO-III), and colon carcinoma cell line (SW620)—and a normal cell line, human diploid lung fibroblast (WI-38).

## 2. Results

### 2.1. Extraction Yield and Tyrosinase Inhibitory Activity of *M. zapota* Bark

The dried bark of *M. zapota* was extracted with *n*-hexane, ethyl acetate (EtOAc), methanol (MeOH), and water, respectively. The *n*-hexane, EtOAc, MeOH, and aqueous crude extracts gave a yield of 140 g (1.93%), 138 g (1.86%), 819.96 g (11.71%), and 96.79 g (1.38%), respectively. They were screened for tyrosinase inhibitory activity (Table 1). l-DOPA was used as a substrate. The EtOAc crude extract exhibited the highest inhibition of tyrosinase activity (IC_50_ 191.69 ± 6.05 μg/mL), followed by the *n*-hexane, MeOH, and aqueous crude extracts, respectively. Kojic acid and α-arbutin showed the inhibitory effect with IC_50_ values of 41.06 ± 3.38 and 57.54 ± 2.54 μg/mL, respectively. Therefore, tyrosinase inhibitory activity-guided fractionation of *n*-hexane and EtOAc crude extracts was carried out to isolate active tyrosinase inhibitors. 

### 2.2. Identification of Compounds ***1***–***8***

**Figure 1:**
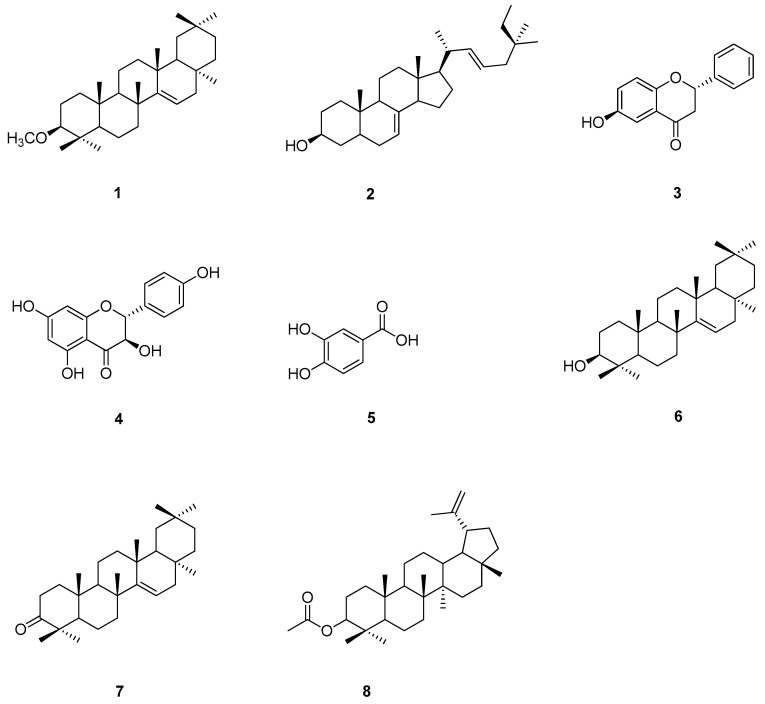
Chemical structures of isolated compounds from *M. zapota* bark: taraxerol methyl ether (**1**); spinasterol (**2**); 6-hydroxyflavanone (**3**); (+)-dihydrokaempferol (**4**); 3,4-dihydroxybenzoic acid (**5**); taraxerol (**6**); taraxerone (**7**); and lupeol acetate (**8**).

The *n*-hexane and EtOAc crude extracts were isolated by using antityrosinase activity-guided fractionation to afford compounds **1**–**8** (Figure 1). Structure elucidation of compounds **1**–**8** was identified by one-dimensional (1D) and two-dimensional (2D) nuclear magnetic resonance (NMR) spectroscopy and high resolution electrospray ionization mass spectrometry (HR-ESI-MS) data and by comparing their spectroscopic data with data found in the literature. Compound **1** was obtained from *n*-hexane crude extract and was identified as taraxerol methyl ether (**1**) [34,35,36]. Compounds **2**–**8** were obtained from EtOAc crude extract and were characterized as spinasterol (**2**), 6-hydroxyflavanone (**3**), (+)-dihydrokaempferol (**4**), 3,4-dihydroxybenzoic acid (**5**), taraxerol (**6**), taraxerone (**7**), and lupeol acetate (**8**) [29,37,38,39,40,41,42,43,44,45].

### 2.3. Spectroscopic Data of Compounds ***1***–***8***

Taraxerol methyl ether (**1**): White amorphous powder (0.47% *w*/*w* of *n*-hexane crude extract), C_31_H_52_O; HR-ESI-MS *m*/*z*: 463.3249 [M + Na]^+^ (calcd. for C_31_H_52_ONa, 463.3916); m.p. 278–280 °C; [α]${}_{D}^{28}$ + 8.253 (c 0.6251; CHCl_3_); Proton nuclear magnetic resonance (^1^H-NMR) (300 MHz, CDCl_3_): δ 5.51 (1H, dd, *J* = 8.1, 3.3 Hz, H-15), 3.33 (s, H-1′), 2.61 (1H, dd, *J* = 11.7, 4.2 Hz, H-3), 2.00 (1H, dt, *J* = 12.0, 3.0 Hz, H-19a), 1.89 (1H, dd, *J* = 15.0, 3.0 Hz, H-1a), 1.65 (1H, m, H-7a), 1.64 (1H, m, H-7b), 1.60 (1H, m, H-1b), 1.57 (2H, m, H-6a and H-21a), 1.51 (2H, m, H-11a and H-21b), 1.44 (1H, m, H-11b), 1.42 (1H, m, H-9), 1.39 (1H, m, H-18), 1.37 (1H, m, H-6b), 1.36 (2H, m, H-12a and H-22a), 1.34 (1H, m, H-19b), 1.31 (1H, m, H-16a), 1.25 (2H, m, H-16b and H-22b), 1.06 (3H, s, H-27), 1.00 (1H, m, H-12b), 0.96 (1H, m, H-2a), 0.95 (1H, m, H-2b), 0.94 (3H, s, H-29), 0.93 (3H, s, H-23), 0.90 (3H, s, H-24), 0.89 (6H, s, H-28 and H-30), 0.85 (1H, d, *J* = 3.6 Hz, H-5), 0.80 (3H, s, H-26), 0.76 (3H, s, H-25); Carbon-13 nuclear magnetic resonance (^13^C-NMR) (100 MHz, CDCl_3_): δ 158.3 (C-14), 116.8 (C-15), 88.9 (C-3), 56.3 (OCH_3_), 55.9 (C-5), 49.1 (C-18), 48.9 (C-9), 41.5 (C-19), 39.2 (C-8 and C-10), 38.8 (C-4), 37.9 (C-7, C-13 and C-17), 37.8 (C-1), 36.8 (C-16), 35.3 (C-12), 33.8 (C-21), 33.5 (C-29), 33.2 (C-22), 30.1 (C-28), 30.0 (C-26), 28.9 (C-20), 28.2 (C-2 and C-23), 26.1 (C-27), 21.5 (C-30), 18.8 (C-6), 17.7 (C-11), 16.3 (C-25), 15.5 (C-24).

Spinasterol (**2**): Colorless crystal (0.0029% *w*/*w* of EtOAc crude extract), C_29_H_48_O; HR-ESI-MS *m*/*z*: 434.3519 [M + Na]^+^ (calcd. for C_29_H_48_ONa, 434.3525); m.p. 166–168 °C; [α]${}_{D}^{28}$ + 2.235 (c 1.7550; CHCl_3_); ^1^H-NMR (300 MHz, CDCl_3_): δ 5.16 (2H, dd, *J* = 15.0, 8.7 Hz, H-7 and H-22), 5.02 (1H, dd, *J* = 15.0, 8.4 Hz, H-23), 3.59 (1H, m, H-3), 2.05 (2H, m, H-12a and H-20), 1.83 (1H, m, H-1a), 1.80 (2H, d, *J* = 3.9 Hz, H-2a and H-14), 1.77 (1H, m, H-6a), 1.74 (1H, m, H-16a), 1.73 (1H, m, H-3a), 1.40 (1H, m, H-5), 1.66 (1H, m, H-9), 1.56 (1H, m, H-11a), 1.55 (1H, m, H-24), 1.53 (1H, m, H-25), 1.49 (1H, m, H-15a), 1.48 (1H, m, H-15b), 1.45 (1H, m, H-11b), 1.43 (1H, m, H-28a), 1.39 (1H, m, H-2b), 1.29 (3H, m, H-3b, H-16b and H-17), 1.26 (1H, m, H-6b), 1.25 (1H, m, H-12b), 1.18 (1H, m, H-28b), 1.08 (1H, m, H-1b), 1.02 (3H, d, *J* = 6.6 Hz, H-21), 0.84 (3H, d, *J* = 2.1 Hz, H-26), 0.81 (3H, s, H-19), 0.80 (3H, d, *J* = 1.8 Hz, H-29), 0.79 (3H, d, *J* = 2.4 Hz, H-27), 0.55 (3H, s, H-18); ^13^C-NMR (100 MHz, CDCl_3_): δ 139.6 (C-8), 138.2 (C-22), 129.5 (C-23), 117.5 (C-7), 71.1 (C-3), 55.9 (C-17), 55.1 (C-14), 51.3 (C-24), 49.5 (C-9), 43.3 (C-13), 40.8 (C-20), 40.3 (C-5), 39.5 (C-12), 38.0 (C-4), 37.1 (C-1), 34.2 (C-10), 31.9 (C-25), 31.5 (C-2), 29.6 (C-6), 28.5 (C-16), 25.4 (C-28), 23.0 (C-15), 21.6 (C-11), 21.4 (C-21), 21.1 (C-26), 19.0 (C-27), 13.1 (C-19), 12.3 (C-29), 12.1 (C-18).

6-Hydroxyflavanone (**3**): Yellow amorphous powder (0.0059% *w*/*w* of EtOAc crude extract), C_15_H_12_O_3_; HR-ESI-MS *m*/*z*: 263.0685 [M + Na]^+^ (calcd. for C_15_H_12_O_3_Na, 263.0684); m.p. 180–182 °C; [α]${}_{D}^{28}$ − 1.1810 (c 1.0500; dimethyl sulfoxide (DMSO)); ^1^H-NMR (300 MHz, (CD_3_)_2_CO): δ 7.53 (1H, dd, *J* = 8.1, 1.5 Hz, H-2′ and H-6′), 7.23 (1H, d, *J* = 3.3 Hz, H-5), 7.06 (1H, dd, *J* = 9.0, 3.3 Hz, H-7), 6.96 (1H, d, *J* = 9.0, 3.3 Hz, H-8), 5.48 (1H, d, *J* = 13.2, 3.0 Hz, H-2), 3.08 (1H, dd, *J* = 16.8, 12.9 Hz, H-3), 2.83 (1H, dd, *J* = 16.8, 3.0 Hz, H-3); ^13^C-NMR (100 MHz, (CD_3_)_2_CO): δ 194.3 (C-4), 156.8 (C-9), 153.1 (C-6), 140.8 (C-1′), 129.7 (C-3′ and C-5′), 129.5 (C-4′), 127.3 (C-2′ and C-6′), 126.0 (C-7), 122.4 (C-10), 120.2 (C-8), 111.5 (C-5), 81.0 (C-2), 45.6 (C-3).

(+)-Dihydrokaempferol (**4**): Yellow amorphous powder (0.0018% *w*/*w* of EtOAc crude extract), C_15_H_12_O_6_; HR-ESI-MS *m*/*z*: 311.0387 [M + Na]^+^ (calcd. for C_15_H_12_O_6_Na, 311.0532); m.p. 228–230 °C; [α]${}_{D}^{28}$ + 26.3548 (c 0.1229; CH_3_OH); ^1^H-NMR (300 MHz, (CD_3_)_2_CO): δ 11.67 (1H, s, OH), 7.40 (2H, d, *J* = 8.4 Hz, H-2′ and 6′), 6.89 (2H, d, *J* = 8.7 Hz, H-3′ and 5′), 5.98 (2H, d, *J* = 2.1 Hz, H-8), 5.94 (2H, d, *J* = 2.1 Hz, H-6), 5.08 (1H, d, *J* = 11.4 Hz, H-2), 4.64 (1H, d, *J* = 11.7 Hz, H-3); ^13^C-NMR (100 MHz, (CD_3_)_2_CO): δ 198.3 (C-4), 167.8 (C-7), 165.0 (C-5), 164.2 (C-9), 158.8 (C-4′), 130.3 (C-2′ and 6′), 129.1 (C-1′), 115.9 (C-3′ and 5′), 101.5 (C-10), 97.0 (C-8), 96.0 (C-6), 84.3 (C-2), 73.1 (C-3).

3,4-Dihydroxybenzoic acid (**5**): Brown amorphous powder (0.0444% *w*/*w* of EtOAc crude extract), C_7_H_6_O_4_; HR-ESI-MS *m*/*z*: 177.0125 [M + Na]^+^ (calcd. for C_7_H_6_O_4_Na, 177.0164); m.p. 220-222 °C; [α]${}_{D}^{28}$ + 13.3030 (c 1.3330; CH_3_COCH_3_); ^1^H-NMR (300 MHz, (CD_3_)_2_CO): δ 7.52 (1H, d, *J* = 2.1 Hz, H-2), 7.47 (1H, dd, *J* = 8.1, 1.8 Hz, H-6) 6.89 (1H, d, *J* = 8.4 Hz, H-5); ^13^C-NMR (100 MHz, (CD_3_)_2_CO): δ 167.5 (C-1′), 150.7 (C-4), 145.6 (C-3), 123.6 (C-6), 123.1 (C-1), 117.5 (C-2), 115.6 (C-5).

Taraxerol (**6**): White crystal (0.0078% *w*/*w* of EtOAc crude extract), C_30_H_50_O; HR-ESI-MS *m*/*z*: 449.3748 [M + Na]^+^ (calcd. for C_30_H_50_ONa, 449.3759); m.p. 284–286 °C; [α]${}_{D}^{28}$ + 0.7210 (c 3.2350; CHCl_3_); ^1^H-NMR (300 MHz, CDCl_3_): δ 5.53 (1H, dd, *J* = 8.1, 3.3 Hz, H-15), 3.19 (1H, dd, *J* = 8.1, 3.3 Hz, H-3), 2.03 (1H, dt, *J* = 11.7, 3.0 Hz, H-19a), 1.92 (1H, dd, *J* = 14.7, 3.0 Hz, H-16a), 1.65 (2H, m, H-2a and H-21a), 1.64 (2H m, H-6a and H-11a), 1.62 (2H, m, H-1a and H-22a), 1.58 (3H, m, H-1b, H-6b and H-21b), 1.44 (1H, m, H-18), 1.39 (1H, m, H-11b), 1.38 (1H, m, H-22b), 1.33 (1H, m, H-19b), 1.31 (2H, m, H-7a and H-12a), 1.30 (1H, m, H-16b), 1.25 (1H, m, H-2b), 1.09 (3H, s, H-27), 1.02 (3H, m, H-7b, H-9 and H-12b), 0.98 (3H, s, H-23), 0.95 (3H, s, H-29), 0.93 (3H, s, H-24), 0.91 (6H, s, H-26 and H-30), 0.82 (3H, s, H-28), 0.80 (3H, s, H-25), 0.76 (1H, d, *J* = 2.7 Hz, H-5); ^13^C-NMR (100 MHz, CDCl_3_): δ 158.2 (C-14), 117.0 (C-15), 79.2 (C-3), 55.7 (C-5), 49.4 (C-18), 48.9 (C-9), 41.5 (C-19), 39.1 (C-8), 38.9 (C-4), 38.2 (C-17), 37.9 (C-1), 37.7 (C-13), 36.8 (C-16), 35.9 (C-10), 35.3 (C-7 and C-12), 33.8 (C-21), 33.5 (C-29), 33.2 (C-22), 30.1 (C-28), 30.0 (C-26), 29.0 (C-20), 28.1 (C-23), 27.3 (C-2), 26.1 (C-27), 21.5 (C-30), 18.9 (C-6), 17.7 (C-11), 15.6 (C-24 and C-25).

Taraxerone (**7**): White crystal (0.0114% *w*/*w* of EtOAc crude extract), C_30_H_48_O; HR-ESI-MS *m*/*z*: 447.3590 [M + Na]^+^ (calcd. for C_30_H_48_ONa, 447.3603); m.p. 248–250 °C; [α]${}_{D}^{28}$ + 8.1315 (c 1.6001; CHCl_3_); ^1^H-NMR (500 MHz, CDCl_3_): δ 5.56 (1H, dd, *J* = 8.1, 2.1 Hz, H-15), 2.58 (2H, m, H-2a and H-3), 2.33 (1H, ddd, *J* = 15.9, 6.3, 3.3 Hz, H-2b), 2.31 (1H, m, H-21a), 2.08 (1H, dt, *J* = 12.9, 3.3 Hz, H-11a, H-19a), 1.87 (1H, m, H-1a), 1.65 (2H, m, H-7a and H-12a), 1.59 (1H, m, H-6a), 1.58 (1H, m, H-11b), 1.50 (3H, m, H-6b, H-9 and H-18), 1.37 (2H, m, H-16a and H-22a), 1.33 (3H, m, H-5, H-16b and H-19b), 1.32 (3H, m, H-22b, H-7b and H-12b), 1.14 (3H, s, H-27), 1.08 (6H, s, H-23 and H-25), 1.06 (3H, s, H-24), 0.99 (2H, m, H-1b and H-21b), 0.95 (3H, s, H-29), 0.91 (6H, s, H-28 and H-30), 0.83 (3H, s, H-26); ^13^C-NMR (100 MHz, CDCl_3_): δ 217.6 (C-3), 157.6 (C-14), 117.3 (C-15), 55.9 (C-5), 48.9 (C-18), 48.8 (C-9), 47.7 (C-4), 40.8 (C-19), 39.0 (C-8), 38.5 (C-1), 37.9 (C-13 and C-17), 37.7 (C-10), 36.8 (C-16), 35.2 (C-7 and C-12), 34.3 (C-2), 33.7 (C-21), 33.5 (C-29), 33.2 (C-22), 30.1 (C-28), 30.0 (C-26), 28.9 (C-20), 26.2 (C-23), 25.7 (C-27), 21.6 (C-24), 21.5 (C-30), 20.1 (C-6), 17.6 (C-11), 14.9 (C-25).

Lupeol acetate (**8**): White amorphous powder (0.0148% *w*/*w* of EtOAc crude extract), C_32_H_52_O_2_; HR-ESI-MS *m*/*z*: 491.3840 [M + Na]^+^ (calcd. for C_32_H_52_O_2_Na, 491.3866); m.p. 210–212 °C; [α]${}_{D}^{28}$ + 45.1328 (c 1.7550; CHCl_3_); ^1^H-NMR (500 MHz, CDCl_3_): δ 4.68 (1H, d, *J* = 1.5 Hz, H-29a), 4.58 (1H, dd, *J* = 1.2, 0.6 Hz, H-29b), 4.47 (1H, dd, *J* = 6.3, 3.6 Hz, H-3), 2.37 (1H, dt, *J* = 9.6, 3.6 Hz, H-19), 2.04 (3H, s, H-2′), 1.91 (2H, m, H-21a and H-30a), 1.67 (1H, m, H-15a), 1.62 (2H, m, H-2a and H-12a), 1.59 (1H, m, H-1a), 1.51 (1H, m, H-6a), 1.49 (1H, m, H-16a), 1.41 (2H, m, H-2b and H-11a), 1.40 (2H, m, H-16b and H-22a), 1.39 (1H, m, H-6b), 1.38 (1H, m, H-7a), 1.35 (1H, m, H-7b), 1.34 (1H, m, H-18), 1.30 (1H, m, H-9), 1.25 (2H, m, H-21b and H-30b), 1.21 (2H, m, H-11b and H-22b), 1.06 (1H, m, H-12b), 1.03 (3H, s, H-23), 0.98 (1H, m, H-13), 0.96 (1H, m, H-1b), 0.94 (3H, s, H-27), 0.86 (1H, m, H-15b), 0.85 (3H, s, H-26), 0.84 (3H, s, H-25), 0.83 (3H, s, H-24), 0.81 (1H, dd, *J* = 4.5, 2.7 Hz, H-5), 0.78 (3H, s, H-28); ^13^C-NMR (100 MHz, CDCl_3_): δ 171.4 (C-1′), 151.1 (C-20), 109.5 (C-29), 81.2 (C-3), 55.5 (C-5), 50.5 (C-9), 48.4 (C-19), 48.2 (C-18), 43.1 (C-14 and C-17), 41.0 (C-8), 40.1 (C-22), 38.5 (C-1), 38.2 (C-13), 37.9 (C-4), 37.2 (C-10), 35.7 (C-16), 34.4 (C-7), 30.0 (C-21), 28.1 (C-23), 27.6 (C-15), 25.2 (C-12), 23.9 (C-2), 21.5 (C-2′), 21.1 (C-11), 19.4 (C-30), 18.3 (C-6), 18.1 (C-28), 16.6 (C-26), 16.3 (C-24), 16.1 (C-25), 14.6 (C-27).

### 2.4. Mushroom Tyrosinase Inhibitory Activity of Compounds ***1***–***8***

Compounds **1**–**8** were tested for their in vitro tyrosinase inhibitory activity (Table 2). l-Tyrosine was used as a substrate for monophenolase inhibitory activity, and l-DOPA was used as a substrate for diphenolase inhibitory activity. In addition, the tyrosinase inhibitory activity of kojic acid and α-arbutin, commercial skin-whitening agents, was also tested for the sake of comparison. (+)-Dihydrokaempferol (**4**) exhibited stronger inhibitory activity (IC_50_ 45.35 ± 0.60 μM) than both kojic acid (IC_50_ 58.53 ± 0.35 µM) and α-arbutin (IC_50_ 353.53 ± 0.55 µM) on monophenolase activity (Table 2). 6-Hydroxyflavanone (**3**) (IC_50_ 53.55 ± 0.45 µM) exhibited monophenolase inhibitory activity similar to that of kojic acid. The other compounds, taraxerol methyl ether (**1**), 3,4-dihydroxybenzoic acid (**5**), taraxerol (**6**), taraxerone (**7**), and lupeol acetate (**8**), showed stronger monophenolase inhibitory activity than α-arbutin (IC_50_ 353.53 ± 0.55 µM), with IC_50_ values of 325.55 ± 0.45, 64.54 ± 0.65, 255.32 ± 0.15, 75.45 ± 0.44, and 155.66 ± 0.51 μM, respectively. Spinasterol (**2**) (IC_50_ 722.44 ± 0.48 µM) showed very weak tyrosinase inhibitory activity.

In this study, the strength of the diphenolase inhibitory activity of the isolated compounds was in the following order: (+)-dihydrokaempferol (**4**) > 6-hydroxyflavanone (**3**) > 3,4-dihydroxybenzoic acid (**5**) > taraxerone (**7**) > lupeol acetate (**8**) **>** taraxerol (**6**) > taraxerol methyl ether (**1**) > spinasterol (**2**). (+)-Dihydrokaempferol (**4**) (IC_50_ 55.41 ± 0.33 μM) displayed similar tyrosinase inhibition to kojic acid (IC_50_ 53.43 ± 0.38 μM) and showed stronger tyrosinase inhibitory activity than α-arbutin (IC_50_ 365.93 ± 0.45 μM) (Table 2).

### 2.5. Kinetic Inhibition of Compounds ***1***–***8*** on Tyrosinase Inhibitory Activity

**Figure 2:**
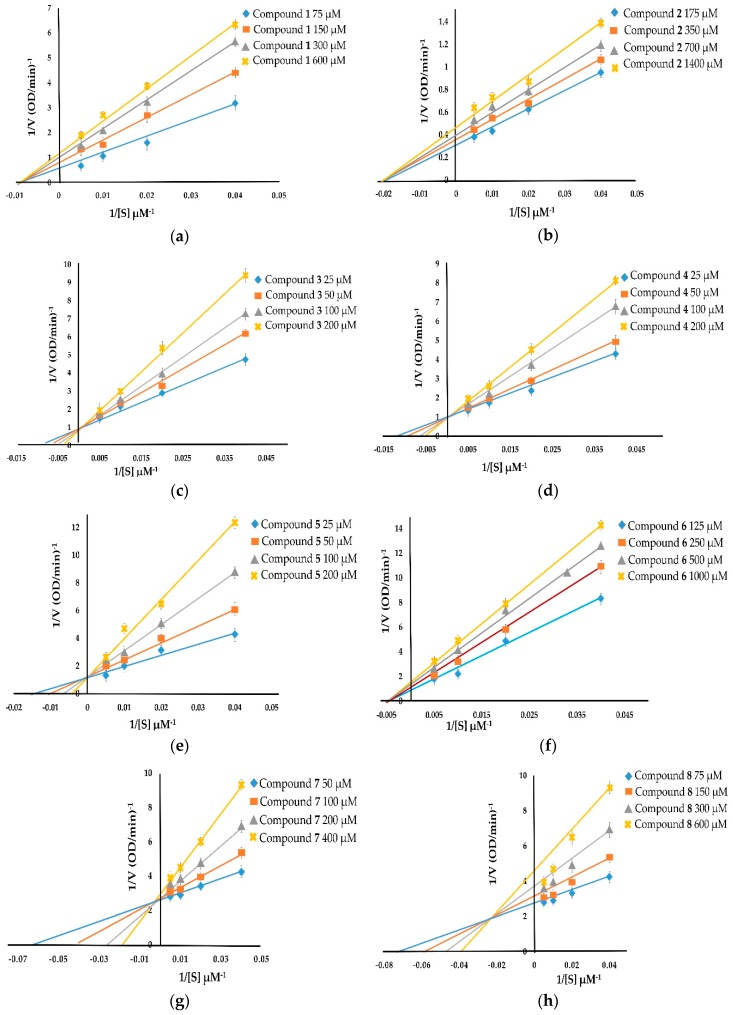
Lineweaver–Burk plots of compounds **1**–**8** showing monophenolase inhibitory activity: (**a**) taraxerol methyl ether (**1**); (**b**) spinasterol (**2**); (**c**) 6-hydroxyflavanone (**3**); (**d**) (+)-dihydrokaempferol (**4**); (**e**) 3,4-dihydroxybenzoic acid (**5**); (**f**) taraxerol (**6**); (**g**) taraxerone (**7**); and (**h**) lupeol acetate (**8**).

**Figure 3:**
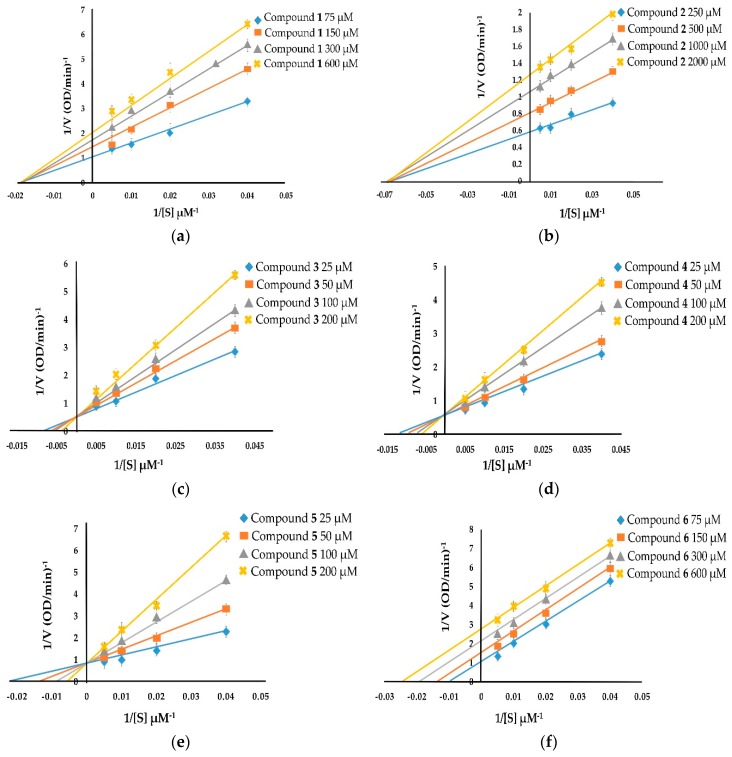

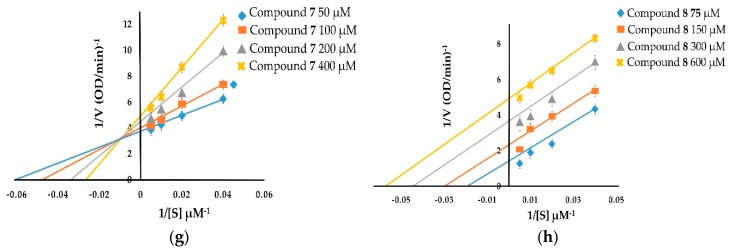
Lineweaver–Burk plots of compounds **1**-**8** showing diphenolase inhibitory activity: (**a**) taraxerol methyl ether (**1**); (**b**) spinasterol (**2**); (**c**) 6-hydroxyflavanone (**3**); (**d**) (+)-dihydrokaempferol (**4**); (**e**) 3,4-dihydroxybenzoic acid (**5**); (**f**) taraxerol (**6**); (**g**) taraxerone (**7**); and (**h**) lupeol acetate (**8**).

The kinetic inhibition of compounds **1**–**8** was determined with respect to both monophenolase and diphenolase activities (Figure 2 and Figure 3). The double-reciprocal plots of 1/V versus 1/[S] showed straight lines with individual slopes and the same horizontal-axis intercept (Figure 2a,b,f). The results indicate that the inhibitor affected the velocity of reaction, but it did not affect the enzyme–substrate complex. It was determined that taraxerol methyl ether (**1**), spinasterol (**2**), and taraxerol (**6**) were noncompetitive inhibitors. The Lineweaver–Burk plots of 1/V versus 1/[S] show straight lines with different slopes and a fixed interception at the Y axis (Figure 2c–e). The results indicate that 6-hydroxyflavanone (**3**), (+)-dihydrokaempferol (**4**), and 3,4-dihydroxybenzoic acid (**5**) were competitive inhibitors. The plots of 1/V versus 1/[S] show a family of straight lines that intersect on the left of the vertical axis (Figure 2g,h). The results demonstrate that taraxerone (**7**) and lupeol acetate (**8**) were mixed inhibitors.

With respect to diphenolase inhibitory activity (Figure 3), taraxerol methyl ether (**1**) and spinasterol (**2**) were noncompetitive inhibitors. Their Lineweaver–Burk plots show a family of lines with different slopes in which the *V*_max_ values were altered, whereas the K_m_ value persisted with the increasing concentration of the inhibitors (Figure 3a,b). 6-Hydroxyflavanone (**3**), (+)-dihydrokaempferol (**4**), and 3,4-dihydroxybenzoic acid (**5**) inhibited diphenolase activity in a competitive manner. The values of K_m_ enlarged with the increase of the inhibitors’ concentration, and the value of *V*_max_ did not change (Figure 3c–e). Taraxerol (**6**) and lupeol acetate (**8**) were uncompetitive inhibitors. Their Lineweaver–Burk plots (Figure 3f,h) show that both the *V*_max_ and K_m_ values were altered with the increasing concentration of taraxerol (**6**) and lupeol acetate (**8**). The relationship between plots of 1/*V* and 1/[S] of taraxerone (**7**) show a family of straight lines that intersect on the left of the vertical axis (Figure 3g). The results demonstrate that taraxerone (**7**) was a mixed inhibitor. This is because taraxerone (**7**) can bind to free enzymes as well as to enzyme–substrate complexes.

### 2.6. Antioxidant Activities of Compounds ***1***–***8***

The antioxidant activities of the isolated compounds **1**–**8** were determined using three different assays, namely 2,2-diphenyl-1-picrylhydrazyl (DPPH) radical scavenging, 2,2′-azino-bis(3-ethylbenzothiazoline-6-sulfonic acid (ABTS) radical scavenging, and ferric reducing antioxidant power (FRAP) assays (Table 3).

#### 2.6.1. DPPH Radical Scavenging Activity

The DPPH radical scavenging activity of compounds **1**–**8** was expressed as 50% of inhibitory concentration (IC_50_) (Table 3). (+)-Dihydrokaempferol (**4**) (IC_50_ 2.21 ± 0.77 μM) showed the highest scavenging DPPH capacity among the isolated compounds. Moreover, 6-hydroxyflavanone (**3**) (IC_50_ 3.21 ± 0.70 μM) and 3,4-dihydroxybenzoic acid (**5**) (IC_50_ 4.71 ± 0.10 μM) exhibited stronger DPPH radical scavenging capacity than taraxerol methyl ether (**1**), spinasterol (**2**), taraxerol (**6**), taraxerone (**7**), and lupeol acetate (**8**), with IC_50_ values of 77.31 ± 0.60, 93.10 ± 0.84, 16.28 ± 0.33, 10.20 ± 0.40, and 87.10 ± 0.31 μM, respectively. The IC_50_ value of the standard Trolox was 1.92 ± 0.22 μM.

#### 2.6.2. ABTS Radical Scavenging Activity

The ABTS radical scavenging ability of compounds **1**–**8** was investigated as IC_50_ (Table 3). The ABTS radical scavenging activity of compounds **1**–**8** decreased in the following order: (+)-dihydrokaempferol (**4**) > 6-hydroxyflavanone (**3**) > 3,4-dihydroxybenzoic acid (**5**) > taraxerone (**7**) > taraxerol methyl ether (**1**) > taraxerol (**6**) > lupeol acetate (**8**) > spinasterol (**2**). The positive control, Trolox exhibited more potent ABTS radical scavenging ability than any of the compounds **1**–**8**.

#### 2.6.3. FRAP Activity

The reducing capacity of compounds **1**–**8** was observed using FRAP assay (Table 3). Among the isolated compounds, (+)-dihydrokaempferol (**4**) was the most potent compound, which expressed a FRAP value of 6.23 ± 0.10 μM. The FRAP value of (+)-dihydrokaempferol (**4**) was slightly similar to the FRAP value of standard Trolox (FRAP value 6.10 ± 0.28 μM). However, 6-hydroxyflavanone (**3**) (FRAP value 4.12 ± 0.12 μM) and 3,4-dihydroxybenzoic acid (**5**) showed higher FRAP values than taraxerol methyl ether (**1**), spinasterol (**2**), taraxerol (**6**), taraxerone (**7**), and lupeol acetate (**8**).

### 2.7. Cytotoxicity of Compounds ***1***–***8***

The cytotoxicity of compounds **1**–**8** and doxorubicin was evaluated using 3-(4,5-dimethylthiazol-2-yl)-2,5-diphenyltetrazolium bromide (MTT) assay on five human carcinoma cell lines, including BT474, Chago-K1, HepG2, KATO-III, and SW620, and compared with human normal cell line WI-38 (Table 4). Spinasterol (**2**) and (+)-dihydrokaempferol (**4**) showed strong cytotoxicity against all the tested carcinoma cell lines. 6-Hydroxyflavanone (**3**) exhibited moderate cytotoxic activity against all the tested carcinoma cell lines. 3,4-Dihydroxybenzoic acid (**5**) displayed moderate cytotoxicity against the BT474 and Chago-K1 cell lines. Taraxerone (**7**) showed strong cytotoxicity against the BT474, Chago-K1, HepG2 and KATO-III cell lines but no cytotoxicity against the SW620 cell line. Lupeol acetate (**8**) exhibited cytotoxicity against only the BT474 cell line. Taraxerol methyl ether (**1**) and taraxerol (**6**) showed weak cytotoxic activity in the all tested carcinoma cell lines. Only spinasterol (**2**) exhibited cytotoxicity against the WI-38 lung fibroblast. Doxorubicin, an anticancer drug, showed potent cytotoxicity in the five human carcinoma cell lines but was non-toxic in the normal cell line.

## 3. Discussion

The bioactivity-guided fractionation of tyrosinase inhibitors from *n*-hexane and ethyl acetate crude extracts of *M. zapota* bark resulted in taraxerol methyl ether (**1**), spinasterol (**2**), 6-hydroxyflavanone (**3**), (+)-dihydrokaempferol (**4**), 3,4-dihydroxybenzoic acid (**5**), taraxerol (**6**), taraxerone (**7**), and lupeol acetate (**8**). Spinasterol (**2**), 6-hydroxyflavanone (**3**), and 3,4-dihydroxybenzoic acid (**5**) were isolated for the first time from this plant. Phytochemical and biological studies of *M. zapota* have been reported previously [23,24,25,26,27,28,29,30,31,32,33]. Polyphenolic compounds and flavonoids from fruits, leaves, and seeds of this plant exhibit significant antioxidant activity and cytotoxicity [23,24,25,26,30]. Methyl 4-*O*-galloylchlorogenate and 4-*O*-galloylchlorogenic acid, which are phenolic compounds, were isolated from *M. zapota* fruits. They showed high antioxidant activity and potent cytotoxicity in the human colorectal cancer HCT-116 and SW480 cell lines [23]. Myricitrin, which is a flavonoid *O*-glycoside, was isolated from *M. zapota* leaves, and it exhibited tyrosinase inhibitory activity [33]. Four kinds of chemical structures in the compounds **1**–**8** isolated from *M. zapota* bark were distinguished in this study. Taraxerol methyl ether (**1**), taraxerol (**6**), taraxerone (**7**), and lupeol acetate (**8**) showed triterpenoid core structures; spinasterol (**2**) was determined to have a sterol core structure; 6-hydroxyflavanone (**3**) and (+)-dihydrokaempferol (**4**) were identified as flavonoids; and 3,4-dihydroxybenzoic acid (**5**) was found to be a phenolic compound. The potency of tyrosinase inhibitory activity depends on the presence of a functional group on the core structure of each compound [46,47]. In this study, the presence of a carbonyl group at C-3 in taraxerone (**7**), which has a triterpenoid core structure, led to strong tyrosinase inhibitory activity when compared with taraxerol methyl ether (**1**), taraxerol (**6**), and lupeol acetate (**8**). This indicates that the carbonyl group was important to the inhibition of triterpenoid analogs in tyrosinase function. (+)-Dihydrokaempferol (**4**) was the most potent tyrosinase inhibitor of the compounds isolated from *M. zapota* bark in this study. It exhibited more potent monophenolase inhibitory activity than kojic acid and similar diphenolase inhibitory activity to kojic acid. Based on its structure–activity relationship, (+)-dihydrokaempferol (**4**) showed tyrosinase inhibitory activity that was stronger than that of 6-hydroxyflavanone (**3**), because it has four hydroxyl groups that substitute at the C-3, C-5, C-7 and C-4′ positions of its flavonoid core structure. In contrast, 6-hydroxyflavanone (**3**) showed only one hydroxyl group, which substitutes at the C-6 position of its flavonoid core structure. In addition, dihydromyricetin, a flavonoid from *M. zapota* fruit, has been reported to demonstrate potent antityrosinase (IC_50_ 3.33 μmol/L for l-DOPA as substrate) and antioxidant activities (IC_50_ for DPPH 12.4 μmol/L and ABTS 3.41 μmol/L). Dihydromyricetin has six hydroxyl groups that substitute at the C-3, C-5, C-7, C-3′, C-4′ and C-5′ positions of its flavonoid core structure [48]. The number and location of such hydroxyl substitutions in the flavonoid structure affected tyrosinase function by formimg hydrogen bonds and hydrophobic interaction [46,47,48,49,50,51,52]. 

Furthermore, the kinetic inhibition demonstrated by compounds **1**-**8** occurred in a dose-dependent manner. The inhibition of 6-hydroxyflavanone (**3**), (+)-dihydrokaempferol (**4**), and 3,4-dihydroxybenzoic acid (**5**) was shown to be competitive inhibition of both monophenolase and diphenolase activities. This indicates that competitive inhibitors only bind with free enzymes [50]. Additionally, it has previously been reported that 3,4-dihydroxybenzoic acid (**5**) is also a tyrosinase substrate, but that its K_m_ is lower than l-DOPA; consistent with its characterization as a competitive inhibitor [53]. Taraxerol methyl ether (**1**) and spinasterol (**2**) were determined to be noncompetitive inhibitors with respect to both monophenolase and diphenolase activities. Based on the results, we found that noncompetitive inhibitors depend on the velocity of reaction and bind at different sites on enzymes. Taraxerol (**6**) was a noncompetitive inhibitor with respect to monophenolase inhibitory activity, but it was an uncompetitive inhibitor with respect to diphenolase inhibitory activity. An uncompetitive inhibitor binds only to enzyme–substrate complexes. Taraxerone (**7**) was a mixed inhibitor with respect to both monophenolase and diphenolase activities. These results indicate that taraxerone (**7**) did not bind to the active sites of enzymes. Previous research reported that the inhibitory mechanism is based on several factors, such as the ability to engage in copper chelating, lack of free radical scavenging, and binding of a compound to the active site of an enzyme [54]. Lupeol acetate (**8**) was a mixed inhibitor with respect to monophenolase inhibitory activity, but it was an uncompetitive inhibitor of diphenolase inhibitory activity. A mixed inhibitor binds to free enzymes and enzyme–substrate complexes at separate sites that are not active sites, whereas an uncompetitive inhibitor binds to enzyme–substrate complexes at separate sites but does not bind to free enzymes [54]. 

In this study, 6-hydroxyflavanone (**3**), (+)-dihydrokaempferol (**4**), and 3,4-dihydroxybenzoic acid (**5**) were potent antioxidants. Usually, antioxidants can protect an organism against ROS. 6-Hydroxyflavanone (**3**) and (+)-dihydrokaempferol (**4**) are flavonoids, which can donate hydrogen similar to phenolic compounds, such as 3,4-dihydroxybenzoic acid (**5**). Thus, flavonoids possess free radical scavenging abilities similar to phenolic compounds [55]. The results demonstrated that the compounds with more phenolic hydroxyl groups have more antityrosinase and antioxidant activities. Therefore, these compounds might have the potential to be used in treatment of skin depigmentation via inhibition of tyrosinase activity. Interestingly, (+)-dihydrokaempferol (**4**) also displayed potent cytotoxicity in the carcinoma cell lines tested: BT474, Chago-K1, HepG2, KATO-III, and SW620. Moreover, it was not toxic to the normal cell line, WI-38. These results suggest that (+)-dihydrokaempferol (**4**) might have the potential to be a good candidate for treatment of skin depigmentation. Thus, *M. zapota* bark might be a good potential source of antioxidants and tyrosinase inhibitors for applications in cosmeceutical products. Additionally, the results of this work may be useful in the study of the structure-activity relationships of flavonoids and antityrosinase activity, to guide the synthesis of desirable new compounds which can act as potent tyrosinase inhibitors.

## 4. Materials and Methods

### 4.1. General Experimental Procedures

Thin-layer chromatography (TLC) was performed on pre-coated silica gel 60 F_254_ plates (layer thickness 0.2 mm, Merck, Darmstadt, Germany). TLC spots were visualized by using ultraviolet (UV) light at 254 nm and 5% H_2_SO_4_ in ethanol. Open column chromatography was conducted using silica gel 60 (70–230 mesh, Merck, Darmstadt, Germany) and Sephadex LH-20 (18–111 μm, GE Healthcare Bio-Sciences AB, Uppsala, Sweden). Melting points were assessed using a SMP-11 melting point apparatus (Keison Products, Chelmsford, UK). Optical rotations were measured with a Polax-2L polarimeter (Atago Co., Ltd., Tokyo, Japan). UV spectra were recorded on a microplate reader Multiscan GO spectrophotometer (Thermo Fisher Scientific Inc., Vantaa, Finland). NMR spectra were obtained on a Bruker model Fourier 300 spectrophotometer instrument (Bruker Daltonics Inc., Bremen, Germany) with tetramethylsilane (TMS) as the internal standard. The chemical shift values were reported in ppm (δ), and the coupling constant (*J*) was in Hz. HR-ESI-MS data were collected using a Bruker model MICROTOF-QII spectrophotometer (Bruker Daltonics Inc., Bremen, Germany). High-performance liquid chromatography (HPLC) was performed on a Liquid Chromatograph (AS3000, Thermo Fisher Scientific Inc., Vantaa, Finland) and consisted of a model SN4000 vacuum degasser, a model P4000 pump, and a model UV6000 detector (Thermo Fisher Scientific Inc., Vantaa, Finland). Separations by HPLC were performed on an Octadecylsilyl (ODS) Thermo Hypersil Keystone column (250 × 4.6 mm i.d., 5 μm, YMC Co., Kyoto, Japan) equipped with a guard column (20 × 3.0 mm i.d., 3.5 μm, Phenomenex Inc., Torrance, CA, USA).

### 4.2. Chemicals and Reagents

Absolute ethanol, acetone-*d*_6_, α-arbutin, chloroform-*d*, dimethyl sulfoxide (DMSO), disodium dihydrogen phosphate monohydrate, l-DOPA, DPPH, Folin–Ciocalteu’s reagent, kojic acid, sodium dihydrogen phosphate monohydrate, l-tyrosine, and sulfuric acid were purchased from Merck (Darmstadt, Germany). Mushroom tyrosinase and Trolox were purchased from Sigma-Aldrich (St. Louis, MO, USA).

### 4.3. Plant Material

The bark of *M. zapota* was collected from Saraburi Province, Thailand, in May 2013. The voucher specimen Bangkok forest herbarium No. 187749 (BKF No. 187749) was deposited at the Forest Herbarium Department of National Parks, Wildlife, and Plant Conservation, Bangkok, Thailand.

### 4.4. Extraction of *M. zapota* Bark

Fresh *M. zapota* bark (175 kg) was dried in a hot air oven at 60 °C. The dried *M. zapota* bark (7 kg) was ground and then extracted with *n*-hexane (3 × 5 L), EtOAc (3 × 5.5 L), MeOH (3 × 5 L), and water (3 × 3.5 L), respectively, for 72 h at room temperature (37 ± 2 °C). The extract was filtrated and evaporated under reduced pressure to afford *n*-hexane (140 g, yellow-green gum), EtOAc (138 g, dark green gum), MeOH (820 g, dark brown gum), and aqueous (97 g, brown gum) crude extracts. The crude extraction yield was calculated using the following equation:

(1) $$\mathrm{Yield}\;\left(\%\right)=\frac{\mathrm{Weight}\;\mathrm{of}\;\mathrm{crude}\;\mathrm{extract}}{\mathrm{Weight}\;\mathrm{of}\;\mathrm{dried}\;\mathrm{bark}}\;\times \;100.$$

### 4.5. Bioactivity-Guided Isolation and Identification of M. zapota Bark

The *n*-hexane crude extract (135 g) was separated by using silica gel quick column chromatography with a gradient elution of *n*-hexane/EtOAc (100:0, 50:50, and 0:100) and EtOAc/MeOH (95:5) to give four fractions (A–D). Then, fractions A–D were performed on antityrosinase activity. Fraction A (60 g) was further fractionated by silica gel column chromatography and eluted with a gradient system of *n*-hexane/dichloromethane (CH_2_Cl_2_) (100:0, 95:5, 50:50, and 0:100) to give four subfractions (A1–A4). Subfractions A1–A4 were evaluated on antityrosinase activity. Subfraction A2 was recrystallized from a mixture of CH_2_Cl_2_/MeOH (80:20) to afford compound **1** (26.2 mg).

The EtOAc crude extract (135 g) was isolated using silica gel quick column chromatography with a gradient elution of *n*-hexane/EtOAc (100:0, 50:50, and 0:100) and EtOAc/MeOH (95:5) to afford four fractions (E–H). They were evaluated for their anti-tyrosinase activity. Fraction F (20 g) was subjected to a silica gel column chromatography with a gradient elution of *n*-hexane/EtOAc (100:0, 80:20, 70:30, 60:40, 30:70, 20:80, and 0:100) to afford six subfractions (F1–F6). Subfraction F1 (1.26 g) was fractionated using silica gel column chromatography and eluted with a gradient system of *n*-hexane/CH_2_Cl_2_ (100:0, 90:10, 80:20, 70:30, 60:40, 30:70, 10:90, and 0:100) to give six subfractions (F11–F16). Subfraction F12 (115.4 mg) was separated by preparative TLC and eluted with heptane/CH_2_Cl_2_ (70:30) to obtain two subfractions (F121–F122). Subfraction F121 (4.2 mg) was separated by preparative TLC and eluted with heptane/CH_2_Cl_2_ (60:40) to yield compound **2** (4.0 mg). Subfraction F4 (1.43 mg) was loaded to column chromatography on silica gel and eluted with a gradient system of *n*-hexane/EtOAc (100:0, 90:10, 70:30, 50:50, 20:80, and 0:100) to obtain six subfractions (F41–F46). Subfraction F43 (133.7 mg) was chromatographed on silica gel column chromatography and eluted with a gradient system of *n*-hexane/EtOAc (80:20, 70:30, 50:50, 20:80, and 0:100) to yield five subfractions (F431–F435). Subfraction F433 (115.2 mg) was subjected to silica gel column chromatography and eluted by a gradient system of *n*-hexane/EtOAc (100:0, 80:20, 70:30, 50:50, 20:80, and 0:100) to obtain six subfractions (F4331–F4336). Subfraction F4334 (13.2 mg) was obtained by repeated purification by analytical HPLC (C18-ODS column, 250 × 4.6 mm i.d., 5 μm particle size) and eluted with MeOH/H_2_O (isocratic elution, 60:40 *v*/*v*, flow rate 1 mL/min, chromatograms were recorded at 254 nm) to obtain compound **3** (8.0 mg). Subfraction F4335 (12.9 mg) was purified by column chromatography on Sephadex LH-20 with methanol as the eluent to give three subfractions (F43351–F43353). Subfraction F43353 (8.0 mg) was subjected to analytical HPLC (C18-ODS column, 250 × 4.6 mm i.d., 5 μm particle size) and eluted with MeOH/H_2_O (isocratic elution, 66:34 *v*/*v*, flow rate 1 mL/min; chromatograms were recorded at 254 nm) to give compound **4** (2.4 mg). Subfraction F5 (3.8 g) was further separated by silica gel column chromatography and eluted with a gradient system of *n*-hexane/EtOAC (100:0, 90:10, 80:20, 70:30, 60:40, 40:60, 30:70, 20:80, 10:90, and 0:100) to afford nine subfractions (F51–F59). Subfraction F59 (123.9 mg) was subjected to silica gel column chromatography and eluted with a gradient system of *n*-hexane/acetone (100:0, 90:10, 80:20, 70:30, 60:40, 40:60, 20:80, 10:90, and 0:100) to obtain 10 subfractions (F59A–F59J). Subfraction F59F was further purified by recrystallization from a mixture of *n*-hexane/acetone (70:10) to afford compound **5** (60.0 mg). The fraction G (4.75 g) was purified by a silica gel column chromatography with a gradient elution of *n*-hexane/EtOAc (100:0, 50:50, and 0:100) and EtOAc/MeOH (95:5) to obtain five subfractions (G1–G5). Subfraction G3 (201.2 mg) was subjected to silica gel column chromatography and eluted by a gradient system of *n*-hexane/EtOAc (100:0, 80:20, 40:60, and 0:100) to obtain four subfractions (G31–G34). Subfraction G33 (51.1 mg) was further purified using a silica gel column chromatography with a gradient system of *n*-heptane/CH_2_Cl_2_ (90:10, 70:30, and 10:90) to obtain three subfractions (G331–G333). Subfraction G332 was purified by preparative TLC and eluted with petroleum ether/CH_2_Cl_2_ (70:30) to yield compound **6** (10.5 mg). Subfraction G333 was recrystallized from MeOH to obtain compound **7** (15.4 mg). Subfraction G5 (200.3 mg) was further subjected to silica gel column chromatography and eluted by a gradient system of *n*-hexane/EtOAc (70:30, 20:80) to yield two subfractions (G51–G52). Subfraction G51 (122.5 mg) was purified using silica gel column chromatography with a gradient elution of CH_2_Cl_2_/EtOAc (30:70, 10:90) to afford compound **8** (20.0 mg).

### 4.6. Mushroom Tyrosinase Inhibitory Assay

The inhibition of tyrosinase activity was performed by spectrophotometry using a modified method of a previously described procedure [56]. l-Tyrosine and l-DOPA were used as substrates for monophenolase and diphenolase activity, respectively. Briefly, a sample was dissolved in a mixture of DMSO/ethanol (1:4 *v*/*v*). The reaction mixture consisted of 150 µL of 0.2 M sodium phosphate buffer (pH 6.8), 50 µL of sample, and 50 µL of substrate solution (500 μM for l-tyrosine/l-DOPA). The reaction was mixed and was incubated for 10 min at 30 °C. Then, 50 µL of tyrosinase solution (200 U/mL) was added, and absorbance was immediately measured at 490 nm (t = 0 min). The assay mixture was then incubated for 20 min at 30 °C, and absorbance was measured at 490 nm (t = 20 min). Kojic acid and α-arbutin were used as positive controls. The percentage of inhibition of tyrosinase activity was calculated using the following equation:

(2) $$\mathrm{Inhibition}\;\left(\%\right)=\left[\frac{\left(A-B\right)\;–\;\left(C-D\right)}{\left(A-B\right)}\right]\;\times \;100.$$

where A is the difference of the absorbance of the control at t = 0 min and t = 20 min, B is the difference of the absorbance of the blank control at t = 0 min and t = 20 min, C is the difference of the absorbance of the test sample and the positive control at t = 0 min and t = 20 min, and D is the difference of the absorbance of the blank of the test sample and the positive control at t = 0 min and t = 20 min.

### 4.7. Kinetic Analysis of Tyrosinase Inhibitory Activity

The kinetic analysis of tyrosinase inhibitory activity was performed with respect to both monophenolase and diphenolase activities. The concentration ranges of the samples were 20–1000 μM. Both l-tyrosine and l-DOPA were concentrated at 0, 25, 50, 100, and 200 μM, respectively. The inhibitory kinetics of the samples were analyzed using Lineweaver–Burk plots.

### 4.8. Antioxidant Assays

#### 4.8.1. DPPH Radical Scavenging Assay

The DPPH radical scavenging activity was determined by a modified method based on a previously described procedure [57]. Briefly, a solution containing 50 µL of sample (100 mg/mL) was dissolved in DMSO/ethanol (1:4 *v*/*v*) and 150 µL of 0.05 M DPPH solution in methanol. Then, the reaction mixture was mixed and was incubated in the dark for 30 min at 37 °C. The absorbance of the reaction mixture was measured at 517 nm. Trolox was used as a positive control. The DPPH scavenging effect was calculated according to the following equation:

(3) $$\mathrm{DPPH}\;\mathrm{radical}\;\mathrm{scavenging}\;\mathrm{activity}\;\left(\%\right)=\left[1-\frac{\left(A_{s}-A_{b}\right)}{A_{d}}\right]\times \;100.$$

where A_s_ is the absorbance of the sample mixed with DPPH solution, A_b_ is the absorbance of the sample without DPPH solution, and A_d_ is the absorbance of DPPH solution without the sample.

#### 4.8.2. ABTS Radical Scavenging Assay

The ABTS radical scavenging capacity was determined using a modified version of a previously described procedure [58]. The stock solution included 100 mL of 7.0 mM ABTS solution in methanol and 100 mL of 2.4 mM aqueous solution of potassium persulfate. Then, the reaction mixture was left in the dark for 14 h at 37 °C. The solution of 1 mL of ABTS solution was diluted with 60 mL of absolute ethanol to determine an absorbance of 0.700 ± 0.001 units at 734 nm using a spectrophotometer. Next, 500 µL of the sample (100 mg/mL) was reacted with 500 µL of ABTS solution and the absorbance was measured at 734 nm after 7 min of incubation using a spectrophotometer. The results were compared with Trolox as a standard, and the percentage of scavenging activity was calculated according to the following equation:

(4) $$\mathrm{ABTS}\;\mathrm{radical}\;\mathrm{scavenging}\;\mathrm{activity}\;\left(\%\right)=\left[\frac{\left(A_{c}-A_{s}\right)}{A_{c}}\right]\times 100.$$

where A_c_ is the absorbance of ABTS radicals with ethanol and A_s_ is the absorbance of ABTS radicals with the test sample or positive control.

#### 4.8.3. FRAP Assay

FRAP assay was conducted using a modified version of a method originally reported in an earlier study [58]. The FRAP reagent contained 25 mL of 0.3 M acetate buffer (pH 3.6), 2.5 mL of 20 mM ferric chloride solution, and 2.5 mL of 10 mM 2,4,6-tris(2-pyridyl)-1,3,5-triazine and was brought to a final volume of 50 mL using 40 mM HCl solution. Then, the FRAP reagent was put into a water bath for 30 min at 50 °C. Next, 600 μL of FRAP reagent was added to 25 μL of the sample (100 mg/mL). The absorbance was recorded at 595 nm after 4 min of incubation using a spectrophotometer. Trolox was used as a positive control. The ferric reducing capacity was expressed as a ferrous sulphate equivalent.

### 4.9. Cytotoxicity Assay

Cytotoxic activity was evaluated in vitro using the microtitration colorimetric method of MTT reduction [59]. In this study, five human carcinoma cell lines were used, including BT474 (ATCC^®^ HTB20^TM^), Chago-K1 (National Cancer Institute, Thailand), HepG2 (ATCC^®^ HB8065^TM^), KATO-III (ATCC^®^ HTB103^TM^), and SW620 (ATCC^®^ CCL227^TM^). Additionally, human diploid lung fibroblast (WI-38, ATCC^®^ CCL75^TM^) was used as the normal cell line for comparison with the carcinoma cell lines. The culturing of these cell lines was derived in complete medium, including Roswell Park Memorial Institute medium (RPMI-1640), fetal bovine serum (5%, *v*/*v*), 25 mM of 4-(2-hydroxyethyl)-1-piperazineethanesulfonic acid (HEPES), sodium bicarbonate (0.25%, *w*/*v*), and kanamycin (100 μg/mL). Doxorubicin was used as a positive control. Each well plate contained 198 μL of culture medium of cell lines and was incubated with 5% CO_2_ atmosphere for 24 h at 37 °C. Then, the culture cells were treated with 2 μL/well of the sample and incubated for 72 h at 37 °C. MTT solution (2 μL, 5 mg/mL in normal saline) was added into each well, and the plates were incubated for an additional 4 h. The supernatant was aspirated out. After that, a mixture of 25 μL of 0.1 M glycine and 150 μL of DMSO was added. The plates were shaken to dissolve the purple-blue crystal of formazan. Then, the absorbance was determined by a microplate reader at 540 nm. The relative cell survival as a percentage of the control (DMSO), which was set at 100%, was calculated using the following formula:

(5) $$\mathrm{The}\;\mathrm{cell}\;\mathrm{survival}\;\left(\%\right)=\left[\frac{A_{s}}{A_{c}}\right]\;\times \;100.$$

where A_s_ is the absorbance of the test sample and A_c_ is the absorbance of a positive control.

### 4.10. Statistical Analysis

All experiments were repeated in triplicate. All data are expressed as mean ± standard deviation. Statistical analyses were evaluated by GraphPad Prism 6 software (GraphPad Software, San Diego, CA, USA). Differences between treatments means were separated by the Tukey test at a significance level of *p* < 0.05.

## 5. Conclusions

The bark of *M. zapota* was extracted with *n*-hexane, ethyl acetate, methanol, and water, respectively, for the evaluation of antityrosinase activity. The ethyl acetate crude extract displayed the highest antityrosinase activity on diphenolase activity. It indicated that ethyl acetate can be a good solvent to extract active tyrosinase inhibitors from *M. zapota* bark. Tyrosinase inhibitors from *M. zapota* bark were isolated by bioassay-guided fractionation. Taraxerol methyl ether (**1**) was isolated from *n*-hexane crude extract. Spinasterol (**2**), 6-hydroxyflavanone (**3**), (+)-dihydrokaempferol (**4**), 3,4-dihydroxybenzoic acid (**5**), taraxerol (**6**), taraxerone (**7**), and lupeol acetate (**8**) were isolated from ethyl acetate crude extract. Spinasterol (**2**), 6-hydroxyflavanone (**3**) and 3,4-dihydroxybenzoic acid (**5**) were isolated for the first time from this plant. (+)-Dihydrokaempferol (**4**) showed potent inhibition of tyrosinase activity with respect to both monophenolase and diphenolase activity. In addition, it was a competitive inhibitor of both monophenolase and diphenolase activity. It also displayed strong antioxidant effects on DPPH, ABTS, and FRAP activity. Furthermore, (+)-dihydrokaempferol (**4**) exhibited potent cytotoxic activity in the BT474, Chago-K1, Hep-G2, KATO-III, and SW620 cell lines. Interestingly, (+)-dihydrokaempferol (**4**) may have the potential to be used as an anticancer agent and antiaging agent for the protection of cell organisms. Thus, *M. zapota* bark might be a good potential source of antioxidants and tyrosinase inhibitors for application in cosmeceutical products. 

## Figures and Tables

**Table 1:** Tyrosinase inhibitory activity of crude extracts of *M. zapota* bark.

Crude Extract	IC_50_ (μg/mL)
*n*-Hexane	557.03 ± 24.13 ^c^
EtOAc	191.69 ± 6.05 ^b^
MeOH	844.22 ± 26.27 ^d^
Aqueous	1660.24 ± 11.29 ^e^
Kojic acid *	41.06 ± 3.38 ^a^
α-Arbutin *	57.54 ± 2.54 ^a^

* Kojic acid and α-arbutin were used as positive controls. Each value represents the mean ± standard deviation of three independent replicates. Different letters in the same column indicate significant differences (*p* < 0.05) within conditions according to Tukey’s multiple range Test.

**Table 2:** Tyrosinase inhibitory activities of compounds **1**-**8**.

Compound	IC_50_ (μM)	
	Monophenolase Inhibitory Activity	Diphenolase Inhibitory Activity
Taraxerol methyl ether (**1**)	325.55 ± 0.45 ^h^	339.33 ± 0.12 ^g^
Spinasterol (**2**)	722.44 ± 0.48 ^j^	973.50 ± 0.28 ^i^
6-Hydroxyflavanone (**3**)	53.55 ± 0.45 ^b^	69.21 ± 0.58 ^b^
(+)-Dihydrokaempferol (**4**)	45.35 ± 0.60 ^a^	55.41 ± 0.33 ^a^
3,4-Dihydroxybenzoic acid (**5**)	64.54 ± 0.65 ^d^	84.66 ± 0.90 ^c^
Taraxerol (**6**)	255.32 ± 0.15 ^g^	276.56 ± 0.56 ^f^
Taraxerone (**7**)	75.45 ± 0.44 ^e^	95.64 ± 0.45 ^d^
Lupeol acetate (**8**)	155.66 ± 0.51 ^f^	139.99 ± 0.33 ^e^
Kojic acid *	58.53 ± 0.35 ^c^	53.43 ± 0.38 ^a^
α-Arbutin *	353.53 ± 0.55 ^i^	365.93 ± 0.45 ^h^

* Kojic acid and α-arbutin were used as positive controls. Each value represents the mean ± standard deviation of three independent replicates. Different letters in the same column indicate significant differences (*p* < 0.05) within conditions according to Tukey’s multiple range Test.

**Table 3:** Antioxidant activities of compounds **1**–**8**.

Compound	IC_50_ (μM)		FRAP (μM)
	DPPH	ABTS	
Taraxerol methyl ether (**1**)	77.31 ± 0.60 ^f^	520.22 ± 0.30 ^f^	1.31 ± 0.16 ^a^
Spinasterol (**2**)	93.10 ± 0.84 ^h^	921.21 ± 0.42 ^i^	1.54 ± 0.21 ^a^
6-Hydroxyflavanone (**3**)	3.21 ± 0.70 ^b^	225.53 ± 0.95 ^c^	4.12 ± 0.12 ^c^
(+)-Dihydrokaempferol (**4**)	2.21 ± 0.77 ^a^	214.83 ± 0.51 ^b^	6.23 ± 0.10 ^d^
3,4-Dihydroxybenzoic acid (**5**)	4.71 ± 0.10 ^c^	290.14 ± 0.95 ^d^	3.00 ± 0.40 ^b^
Taraxerol (**6**)	16.28 ± 0.33 ^e^	630.84 ± 0.54 ^g^	1.46 ± 0.11 ^a^
Taraxerone (**7**)	10.20 ± 0.40 ^d^	334.83 ± 0.99 ^e^	1.12 ± 0.13 ^a^
Lupeol acetate (**8**)	87.10 ± 0.31 ^g^	669.62 ± 0.42 ^h^	1.28 ± 0.30 ^a^
Trolox *	1.92 ± 0.22 ^a^	188.39 ± 0.43 ^a^	6.10 ± 0.28 ^d^

* Trolox was used as a positive control. DPPH = 2,2-diphenyl-1-picrylhydrazyl, ABTS = 2,2′-azino-bis(3-ethylbenzothiazoline-6-sulfonic, FRAP = ferric reducing antioxidant power. Each value represents the mean ± standard deviation of three independent replicates. Different letters in the same column indicate significant differences (*p* < 0.05) within conditions according to Tukey’s multiple range Test.

**Table 4:** Cytotoxic activity of compounds **1**–**8**.

Compound	IC_50_ (μM)					
	BT474	Chago-K1	HepG2	KATO-III	SW620	WI-38
Taraxerol methyl ether (**1**)	184.95 ± 1.61 ^g^	>227.07 ^h^	>227.07 ^g^	>227.07 ^g^	>227.07 ^f^	>227.07 ^d^
Spinasterol (**2**)	9.16 ± 1.97 ^b^	16.53 ± 2.84 ^c^	10.87 ± 1.12 ^b^	13.73 ± 3.69 ^b^	33.03 ± 2.50 ^b^	9.85 ± 1.90 ^a^
6-Hydroxyflavanone (**3**)	86.16 ± 0.45 ^f^	57.73 ± 1.08 ^e^	65.76 ± 2.37 ^e^	88.78 ± 3.70 ^e^	82.79 ± 1.33 ^d^	>416.22 ^g^
(+)-Dihydrokaempferol (**4**)	11.66 ± 0.42 ^c^	12.32 ± 0.73 ^b^	13.67 ± 0.38 ^c^	39.79 ± 0.38 ^d^	41.11 ± 1.08 ^c^	>346.92 ^h^
3,4-Dihydroxybenzoic acid (**5**)	85.21 ± 3.96 ^f^	79.22 ± 4.02 ^f^	364.72 ± 2.27 ^i^	507.53 ± 4.61 ^i^	591.36 ± 0.71 ^i^	>648.85 ^i^
Taraxerol (**6**)	>235.45 ^h^	>235.45 ^i^	>235.45 ^h^	>235.45 ^h^	>235.45 ^h^	>235.45 ^f^
Taraxerone (**7**)	19.24 ± 0.40 ^d^	26.75 ± 0.97 ^d^	20.41 ± 1.43 ^d^	26.49 ± 0.57 ^c^	>234.34 ^g^	>234.34 ^e^
Lupeol acetate (**8**)	60.20 ± 0.90 ^e^	199.87 ± 0.30 ^g^	>213.33 ^f^	136.68 ± 0.66 ^f^	182.67 ± 1.51 ^e^	>213.33 ^c^
Doxorubicin *	1.21 ± 0.20 ^a^	1.58 ± 0.40 ^a^	2.70 ± 0.83 ^a^	1.78 ± 0.83 ^a^	1.82 ± 0.39 ^a^	>183.99 ^b^

* Doxorubicin was used as a positive control. BT474 = breast carcinoma cell line, Chago-K1 = lung bronchus carcinoma cell line, HepG2 = liver carcinoma cell line, KATO-III = gastric carcinoma cell line, SW620 = colon carcinoma cell line, and WI-38 = human diploid lung fibroblast. Each value represents the mean ± standard deviation of three independent replicates. Different letters in the same column indicate significant differences (*p* < 0.05) within conditions according to Tukey’s multiple range Test.
